# A deep learning-based model for detecting depression in senior population

**DOI:** 10.3389/fpsyt.2022.1016676

**Published:** 2022-11-07

**Authors:** Yunhan Lin, Biman Najika Liyanage, Yutao Sun, Tianlan Lu, Zhengwen Zhu, Yundan Liao, Qiushi Wang, Chuan Shi, Weihua Yue

**Affiliations:** ^1^Institute of Mental Health, Peking University Sixth Hospital, Beijing, China; ^2^Research Unit of Diagnosis and Treatment of Mood Cognitive Disorder, Chinese Academy of Medical Sciences (2018RU006), Beijing, China; ^3^National Clinical Research Center for Mental Disorders and NHC Key Laboratory of Mental Health and (Peking University Sixth Hospital), Beijing, China; ^4^Voice Health Tech, Beijing, China; ^5^The Fifth Hospital of Tangshan City, Tangshan, China; ^6^PKU-IDG/McGovern Institute for Brain Research, Peking University, Beijing, China; ^7^Chinese Institute for Brain Research, Beijing, China

**Keywords:** deep learning, major depressive disorder, screening test, senior population, acoustic information

## Abstract

**Objectives:**

With the attention paid to the early diagnosis of depression, this study tries to use the biological information of speech, combined with deep learning to build a rapid binary-classification model of depression in the elderly who use Mandarin and test its effectiveness.

**Methods:**

Demographic information and acoustic data of 56 Mandarin-speaking older adults with major depressive disorder (MDD), diagnosed with the Mini-International Neuropsychiatric Interview (MINI) and the fifth edition of Diagnostic and Statistical Manual of Mental Disorders (DSM-5), and 47 controls was collected. Acoustic data were recorded using different smart phones and analyzed by deep learning model which is developed and tested on independent validation set. The accuracy of the model is shown by the ROC curve.

**Results:**

The quality of the collected speech affected the accuracy of the model. The initial sensitivity and specificity of the model were respectively 82.14% [95%CI, (70.16–90.00)] and 80.85% [95%CI, (67.64–89.58)].

**Conclusion:**

This study provides a new method for rapid identification and diagnosis of depression utilizing deep learning technology. Vocal biomarkers extracted from raw speech signals have high potential for the early diagnosis of depression in older adults.

## Introduction

Depressive disorder is a common mental disorder that brings great pain to patients and their families, which is a collection of similar signs and symptoms. Depressed patients are in an unpleasant state of mind for a prolonged period time, even if they hear good news or are in a pleasant environment. Lack of interest makes people with depression often avoid socializing, and loss of energy makes it difficult to complete tasks, making it increasingly difficult to work and study. In addition to emotional changes, patients with depressive disorder also show a series of biological symptoms, including sleep disorders characterized by early awakening, diurnal mood changes, changes of appetite, weight loss, decreased libido, and even menopause in female patients (1). This common mental disorder is often under-diagnosed or misdiagnosed due to collection of similar signs and symptoms. According to the report released by the World Health Organization (WHO) in 2017 (2), it is estimated that more than 300 million people worldwide suffer from depression, with an average global incidence of 4.4%, while the lifetime prevalence rate of depression in China is 6.8% (3). The COVID-19 pandemic has intensified adverse social factors, which caused a series of negative effects on public mental health (4–7).

The combination of the symptoms creates unprecedented burden on families which is more severe when the affected patient is an elderly member of the family. With the busy lifestyle of the younger family members and inability to seek timely help due to age, mobility issues etc., elderly patients have much more higher misdiagnosis rates as well as lower chances to receiving adequate support. Recently the diagnosis and treatment of depressive disorder have become the focus of research. The traditional diagnosis of depressive disorder is to conduct interviews and observations of patients with professionally trained psychiatrists, and evaluate whether patients have depressive disorders according to the diagnostic criteria. A shortage of psychiatrists makes effective identification of depressive disorders more difficult. As of 2004, there were only 16,103 licensed psychiatrists in China (1.24 per 100,000 population), and the average global mental health workforce is 4.15 psychiatrists per 100,000 people. Although this number has increased to 36,680 in 2019 (8), it still presents a clear shortage in the face of China's huge population base compared to the average of foreign (9). Meanwhile, current methods of diagnosing of depression rely primarily on self-reports and are therefore often confused by issues such as unawareness of symptom severity, concealment of illness and etc. (10, 11). Successful depression screening and monitoring may provide earlier diagnosis and finer-grained treatment, which may help improve the prognosis of severe depressive disorder.

In recent years, non-invasive and continuous monitoring of physiological and psychological data by smartphone-based technologies and wearable devices has aroused researchers' interests (12). Speech recognition technologies, such as automatic speech recognition and machine translation, has reached the peak. The technology also has a higher adoption rate in elderly community. In the recent years the mobile hardware and mobile operating systems support variety of mobile applications that takes raw audio input and complete tasks such as sending voice messages, important voice commands as well as user identification and authentication. These advancements in acoustic and speech processing makes a new frontier of behavioral health diagnosis using machine learning. It was found that the depressive patients showed reduced stress, mono-pitch, loudness decay and etc. in language, which was consistent with the clinical observations of depressed patients. These patients showed slower speech, more pauses, and low volume compared to ordinary people (13, 14). Studies have shown that the speech biomarkers recognized by machine learning models have a positive correlation and have greater potential in detecting mental disorders such as depression (15–18). Researchers have spent tremendous amount of effort studying depression and its correlation to acoustics and semantic aspects of speech. There is extensive research on detecting anxiety and depression through acoustic and semantic aspects of speech using machine learning techniques (19, 20). Feature extraction has been the most common research direction on designing machine learning systems (13, 21). a range of acoustic features have been identified as predictive of depression, such as rhythmic features, spectral-based correlation features, and phonetic features Advancements in the deep learning techniques have created a new research approach with improved performance (22, 23).

However, most of these studies focus on laboratory data sets recorded in a highly controlled environment based on PHQ-9 or PHQ-8 labeling, limiting their use and potential value in the real world (24). On the other hand, traditional speech and acoustic technologies usually use machine learning models which requires very careful design on hand engineered features and large amount of labeled data (13, 15). Over the past few years, speech-based deep learning technology has shown significant progress, and using deep learning to detect, depression from speech has aroused widespread concern (25). In the recent years deep learning researchers have successfully implemented state of the art results with utilizing self-supervised learning in both computer vision and automatic speech recognition domains, which can learn how to extract latent feature representations from complex, diverse and unlabeled data from many fields. This is an effective method to build a more robust and better classifiers which generalize effectively in downstream tasks (26).

Over the decades the neurological-physiological differences between young and old speakers have been widely studied by researches (27, 28). Meanwhile, the current research on the classification of depression based on speech mainly uses mixed samples from all age groups together, while age is one of the influencing factors of voice. Due to the aging of facial muscles, the loss of teeth, the increase of psychomotor retardation and other reasons, the voice of the elderly is hoarser, and their vocal characteristics are closer to those of patients with depression (28, 29). Compared with young people, judging depression in the elderly based on sound may face greater challenges.

For these reasons, we use Chinese voice data recorded using a variety of smartphones and environments, marked by psychiatrists following DSM-5 criteria standards. This paper shows for the first time the results of an acoustic large-scale depression detection system in the elderly population.

## Materials and methods

### Participants and procedures

For evaluating the model performance an external test set with 109 participants between 50 and 65 years old were recruited for the study. All participants gave informed consent to participate in this investigation. Inclusion criteria were age between 50 and 65 years old, with Han nationality and diagnosed with MDD using DSM-5. Exclusion criteria with one of the following conditions: (1) other mental disorder except depressive disorder and (2) any factors that may change the voice such as influenza or vocal training.

The participants were interviewed by psychiatrists, and voice samples were recorded using smart-phone in the same day, and were divided into disease group and control group according to DSM-5 and MINI. Before the acoustic data collection session, the patients completed the Self-Rating Depression Scale, and were evaluated by MINI, which is the reference standard, as well as Hamilton Rating Scale for Depression (HAMD) framework (roughly 30–90 min sessions). After the interview was concluded the patient's consent is taken to be part of a data collection exercise. Data collection system uses a We-chat mini program to collect audio recording of the participants using their own mobile phone with a 16 kHz sampling rate. Through the mini-program, the participants filled out Medical Record Number (provided by researchers). Participants then received information about the trial, confirmed the informed consent in the applet, and completed the SDS questionnaires. In subsequent sessions, participants are requested to read a fixed Chinese paragraph which is designed by linguistic experts and psychoacoustics experts to capture acoustic and phoneme variations from speech. The recorded data is uploaded to the data management system and a series of automated and human pre-processing is applied to ensure the data quality. Written informed consent was obtained from the individuals for the publication of any potentially identifiable images or data included in this article.

### Deep learning models

We used a corpus of Mandarin Chinese conversation speech dataset Oizys collected by Voice Health Tech annotated partnering with Peking University Sixth Hospital, The Fifth people's Hospital of Tangshan, and Weihai Mental Health Center etc. As open sources speech datasets lack diversity and are usually small especially in the mental health space, Oizys enables us to conduct quantitative and qualitative analysis with considerable statistical significance for both training and evaluation datasets. Our dataset is currently proprietary due to privacy issues and ethical standards associated with health-related data. Our corpus for this experiment contains 1,589 unique speakers, 64.8% of whom are female and 35.2% of whom are male. Users ranged in age from 18 to 70 with an average age of 34. Questions were designed to explore strong signals reflecting a range of acoustic variations.

**Figure 1:**
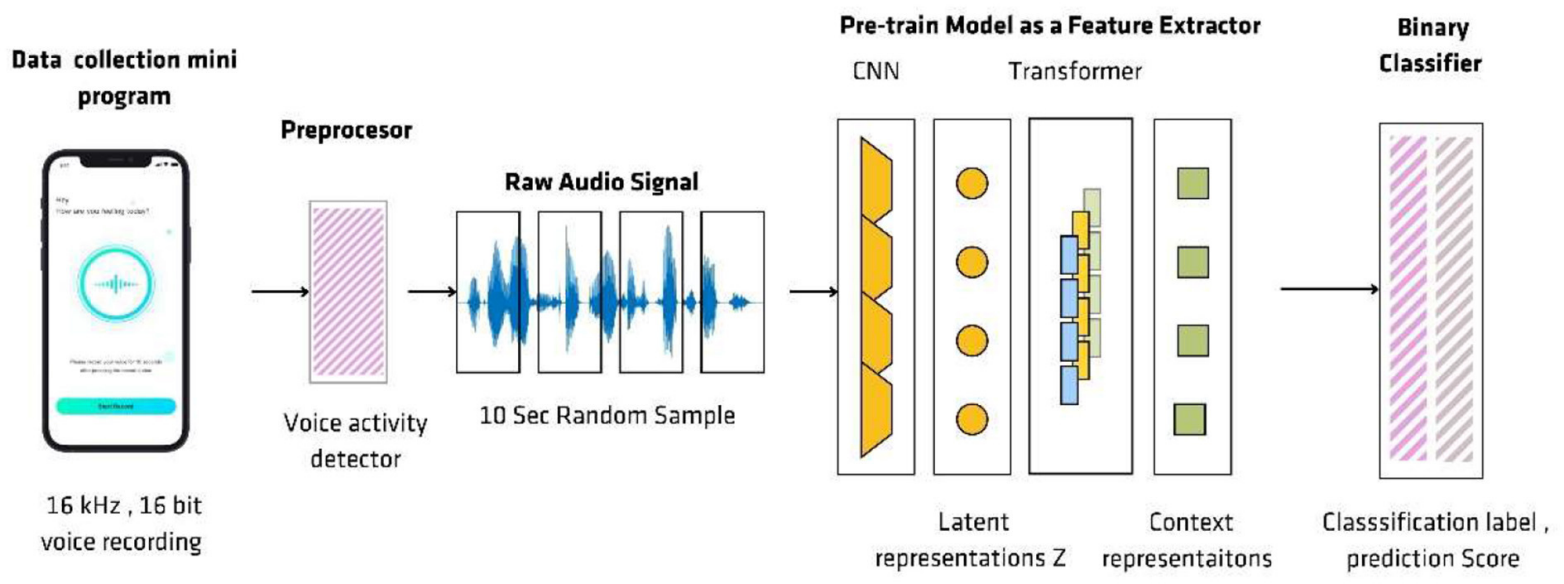
The flow of voice processing. Audio and other metadata are collected *via* a We-chat mini program and pre-processed by voice activity detector to remove silence in the leading and training end of the voice clip. A 10 s random sample is extracted from a preprocessed audio clip and later fed into a pre-trained model to extract features.

Our dataset is then split into an 80% for training and 20% for validation with a speaker independent approach, where validation set would not share samples with speaker from the training set. As shown in Figure 1, the audio data is pre-processed with a pre-processing engine evaluating the recorded files for signal to noise ratio as well as speech rate to ensure quality. It is then sent *via* an automatic transcription software and later cross checked with audio for errors to ensure the quality of the transcribed audio. Then the recorded data is processed through a preprocessing step where a voice activity detector is used to remove silence in the leading and training end of the voice clip to ensure the voiced content of the audio file. In order to ensure the robustness of the data while training a series of data augmentations have been employed such as random sampling, room reverberation addition, noise addition. Furthermore, the signal to noise ratio (SNR) of the dataset is calculated. A sampling strategy is used to balance the SNR levels of depressed and healthy samples in between the classes to ensure both classes have a standard distribution in signal to noise ratio. Finally, a random 10 s sample is extracted from a preprocessed audio clip and later fed into a pre trained model to extract features.

We conduct our experiments on two different models. First, a strong speech baseline model is used for comparing our approach to a traditional deep learning approach without the use of per-training described in “DepAudioNet baseline”. Second, a pertained speech classifier pre-trained on large quantities of speech data in a self-supervised way. We describe this classifier in Section “Pre-trained speech classifier.”

#### DepAudioNet baseline

In order to setup a baseline model for comparison we used DepAudioNet (30), DepAudioNet presents a novel and effective audio-based method on depression classification. DepAudioNet is one of the most popular works in the literature leveraging deep learning for depression detection. The model architecture is composed of a one-dimensional convolutional layer to model short-term dependencies, followed by a one-dimensional max-pooling layer to capture middle-term correlations, and then a recurrent neural network (Long Short-Term Memory) to model longer-term relations in the audio sequence. Finally, the output of the LSTM is fed to two fully connected layers to lead to a single prediction score. A batch normalization layer as well as a ReLU activation are applied between the output of the 1-D convolutional layer and the input of the max-pooling layer. A dropout regularization layer is applied between the max-pooling and LSTM layers.

The model is fed Mel-scale filter banks features, a kind of Low-Level Descriptor (LLD) applied on a small window of audio which was first pre-processed through a silence detector to keep only speech audio.

The optimal hyper-parameters mentioned in DeepAudionet model (30) is reused for our baseline CNN-RNN model: The model architecture consist of a CNN layer which has 128 feature maps with kernel 3 and stride 1 followed by a max-pooling layer also has a kernel size of 3, and the two last fully connected layers have 128 and 1 units, respectively.

Forty Mel-scale filter bank features were extracted from segments of around 3.84 s, using the same parameters as specified in the original paper. In order to remove silences from the audio files before extracting the Mel-scale filter bank features as voice activity detector (rVAD) (30) is used. According to the original paper, the Stochastic Gradient Descent (SGD) algorithm is used for optimization with a batch size of 32 examples.

However, since no mention is made of the learning rate used, how long the network was trained, or what regularization methods were used (such as early stopping), we perform a hyper-parameter search on Oizys development set for a learning rate in 1e−4, 3e−4, 1e−5, dropout of 0.2, 0.4, and we use early stopping based on the class-averaged F1 score (macro F1) on the development set. We report results for our best model on test set in respect to Sensitivity and Specificity and AUC.

#### Pre-trained speech classifier

Pre-trained Speech Classifier used for fine tuning Oizys dataset, consists of an automatic speech recognition model pre-trained on opensource datasets. Nine hundred sixty hours of the Libri speech dataset (31) on 16 kHz audio, as well as the robust version of same model, pre-trained on 19 h of LibriLight, Common Voice (32), Switchboard (33), Fisher (34) in a self-supervised manner is used for loading pre-trained weights and extracting embeddings.

The pretrained model architecture is made of a feature encoder (7-layer convolutional network) to first extract latent features z_0_, z_1_ …z_T_ from raw speech every 20 ms, with a receptive field of 25 ms.These latent features are then sent through a Transformer encode, where each position in the sequence attends to every other position. This allows the Transformer network to produce contextual representations at each timestep c_0_, c_1_...c_T_ that capture long-range dependencies across the entire audio sample. In parallel, the latent features z_t_ are also sent to a quantization module that uses Softmax-Gumbel to transform the se continuous features into quantized representations q_0_, q_1_...q_T_ from a finite set of possible quantized representations.

The model is pre-trained in a self-supervised way, where the final contextual features c_t_ are used to distinguish the real quantized representations qt at masked time steps from false distractors q. Around 50% of the latent features z_t_ are masked, replaced by a unique learnable feature vector z mask, before being sent to the Transformer. Then, a similarity score (cosine similarity) is computed between contextual features c_t_ at masked positions and candidate representations: ground truth qt along with 100 distractors q. A contrastive loss is used for training, encouraging high similarity scores with real targets qt and low similarity with distractors q. An additional diversity loss is also combined with this contrastive loss to push the model to use all possible quantized representations and not just a small subset of it. Altogether, this training objective forces the model to learn information-rich contextual representations c_T_ to be able to properly distinguish a large diversity of targets.

These contextual speech representations from the Transformer encoder are re-used in our depression detection classifier. These embeddings c_0_, c_1_…c_T_ were extracted from a given layer l in the Transformer encoder, and then apply a mean pooling operation to aggregate these contextual representations into a single sequence-level representation. This final representation is then sent to a fully connected neural network to produce a final depression score used for prediction on the given raw audio segment. Similar to the baseline model, predictions from multiple segments were combined in the same audio file with majority voting to calculate final depression prediction for a data sample.

#### Depression classifier

The depression classifier consists of a pretrained model with 317 million parameters. The model architecture is described as follows; 7-layer convolutional network followed by a 24-block Transformer encoder network with embedding dimension 1,024. It has been shown that as layers contain different kinds of information, therefore it is worthwhile to investigate which layers benefit the most for the downstream task of depression classification. The layer with the most discriminative power after training multiple classifiers on embeddings from different layers is selected. A grid search was performed with the following parameters l ∈ [14; 24], learning rate lr ∈ {1e−5, 3e−5, 1e−4}, segment duration d ∈ {5, 10, 15} seconds and optimal parameters was selected for training the depression classifier.

#### Training process

For depression classifier model, random sampling approach (30) was re-used. This simple training strategy alleviates the problem of class imbalance, as healthy individuals outnumbered depressed individuals in the dataset. For each training batch, same amount of audio segments were sampled from both classes. This is similar to an under-sampling strategy for the majority class, as not every healthy patient will be seen during one epoch.

### Statistical methods

The *t*-test (continuous variables) and the χ^2^ and Fisher exact tests (categorical variables) were used to evaluate for demographic and clinical characteristics. The data lacking any diagnostic method is not included in the data analysis, and the missing general demographic data is uniformly recorded as “unknown.” The McNemar test was used to evaluate the proportion difference of classification variables, the Spearman correlation analysis and 2-level logistic regression analysis was to evaluate the stability of the model and SPSS software was used for analysis. The confidence interval is 95%, and Normal Approximation Method is used to estimate the confidence interval of binomial distribution. The hypothesis of this study is that the model can effectively distinguish between patients and healthy controls. The hypothesis is that the prediction efficiency of the model is better when the area under the ROC curve is >0.7. In the previous experiment, the area under the ROC curve of the model was 0.88 when α = 0.05 and β = 0.1, the sample size was estimated by PASS11. The results showed that at least 39 patients and 39 controls were needed. Considering the loss of follow-up rate of 10%, 44 patients and 44 controls were included in the study at least. The existing sample size has met the above requirements.

## Results

### Demographic and clinical characteristic

In order to test the performance of the depression classifier we collected a separate test set containing 109 participants in this trial who were depressed and non-depressed people aged between 50 and 65 collected online and outpatient of Peking University Sixth Hospital, The Fifth people's Hospital of Tangshan from November 2020 to September 2021, of which two were not enrolled because of ethnic minorities and four were excluded because of depression remission, which we'll mention in the following parts. Depressive episode was diagnosed in 56 individuals with 21 males (37.5%) and 35 females (62.5%), while the healthy control consisted of 17 males (36.2%) and 30 females (63.8%). As Table 1 shows, the mean age of disease group is 57.73 years (±4.69), and control group is 57.57 years (±4.96, *p*-value 0.869). There were no significant differences in age, gender and BMI between disease group and healthy control group (*p* > 0.05; Table 1). Among 103 participants, there was a statistically significant difference in the HAMD score, HAMA score, SDS score between the disease group and the control group (*p* < 0.05). The detail of demographic information of participants can be founded in Supplementary material.

**Table 1:** Demographic and clinical characteristic.

**Variable**	**Mean** ±**SD**^**a**^	
	**Disease group (*n* = 56)**	**Control group (*n* = 47)**	***p*-value**	***T*-value/x^2^**	***df***
Age, year^b^	57.73 (±4.69)	57.57 (±4.96)	0.869	−0.166	101
Gender (male/female)^c^	21/35	17/30	0.889	0.019	1
BMI^b^	23.21 (±2.42)	24.08 (±2.46)	0.076	1.792	100
HAMD^b^	19.61 (±6.56)	0.74 (±1.53)	0.000	−20.846	61.994
HAMA^b^	17.14 (±6.92)	0.38 (±1.05)	0.000	−17.869	58.026
SDS^b^	59.38 (±12.09)	32.62 (±8.42)	0.000	−13.182	97.862

a Data are presented as mean ± standard deviation.

b P by two-tailed two-sample T-test.

c P by two-tailed Person Chi-square test.

BMI, Body mass index; HAMD, Hamilton depression rating scale; HAMA, Hamilton anxiety rating scale; SDS, Self-rating depression scale.

### ROC curve analysis

**Figure 2:**
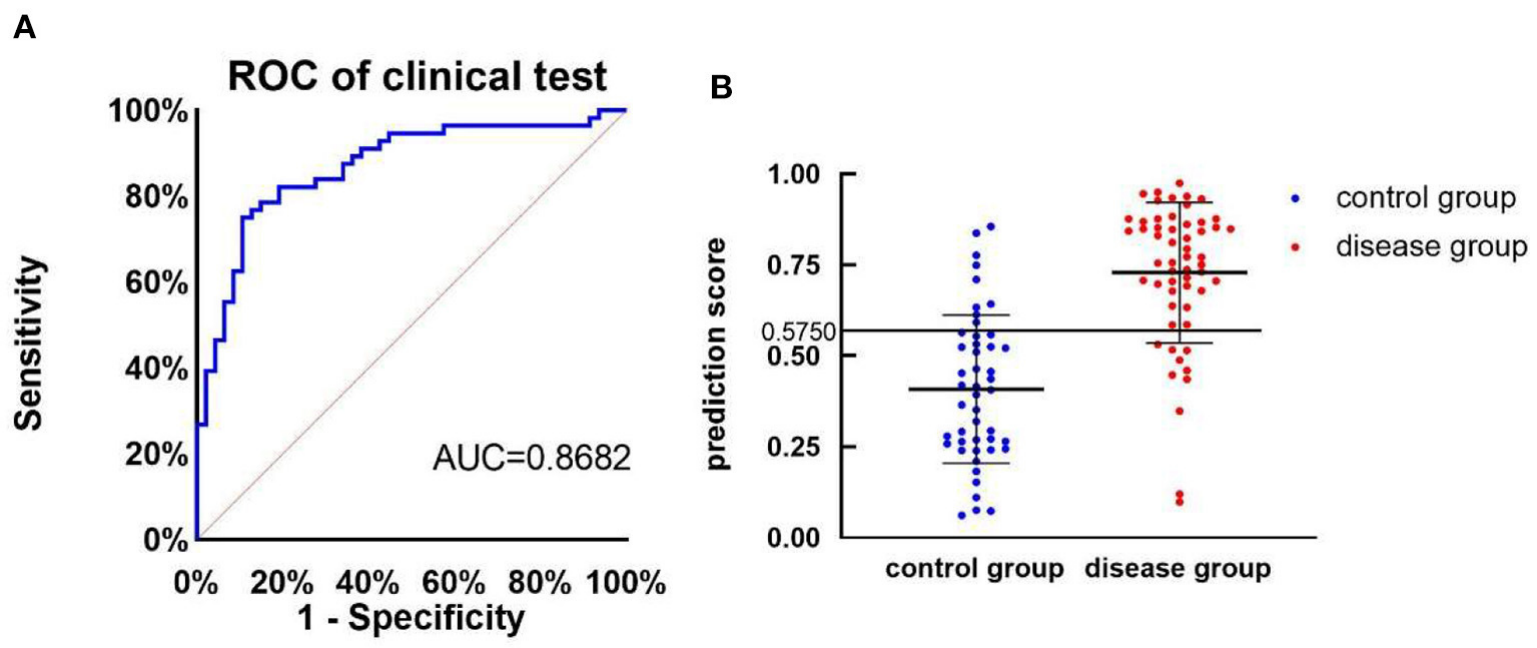
The performance of depression classifier. **(A)** ROC curve of pre-training model of clinical test. **(B)** The prediction score of health controls (*n* = 47, control group) and participants with major depressive disorder (*n* = 56, disease group), AUC, area under the ROC curve, the cut-off is 0.5750 with the sensitivity is 82.14% and specificity is 83.64%.

ROC curve analysis was used to evaluate the 2-level classification ability of deep leaning models. As shown in Figure 2A, the area under the ROC curve is 0.8682. The maximum accuracy rate appears when prediction threshold is 0.6607 with the sensitivity is 75% and specificity is 89.36%. Considering the requirements of the model in the real world, we finally choose to set the cut-off prediction threshold at 0.5750. If the score is >0.5750, the model classifies it as depression. As shown in Figure 2B, the part above the dotted line (prediction threshold ≥0.5750) is judged by the model as depressive disorder.

Of 56 depression diagnosed by DSM-5 and MINI, deep learning model considered 46 individuals as positive outcomes and 10 individuals as negative outcomes [sensitivity, 82.14% (95%CI, 70.16–90.00)]; Of 47 individual without depressive episode, 38 voice specimans was considered as positive results and 9 as negative. [specificity, 80.85% (95%CI, 67.64–89.58)]. The positive predictive value is 83.64% (95%CI, 71.74–91.14) and the negative predictive value is 79.17% (95%CI, 65.74–88.27; *p* < 0.001, Table 2).

**Table 2:** Performance metric sensitivity, specificity.

		**Standard reference**		
		**Disease group^*d*^, *n***	**Control group^*e*^, *n***	**Total, *n***
**AI model** ^**a**^	Positive^b^, *n*	46	9	55
	Negative^c^, *n*	10	38	48
	Total, *n*	56	47	103

a AI model: pre-trained acoustic model.

b Positive: Major depressive disorder classified by computer.

c Negative: Healthy control classified by computer.

d Disease group: participants with Major depressive disorder diagnosed with the Mini-International Neuropsychiatric Interview (MINI) and Diagnostic and Statistical Manual of Mental Disorders, Fifth Edition (DSM-5).

e Control group: participants without Major depressive disorder.

### Baseline comparison

**Figure 3:**
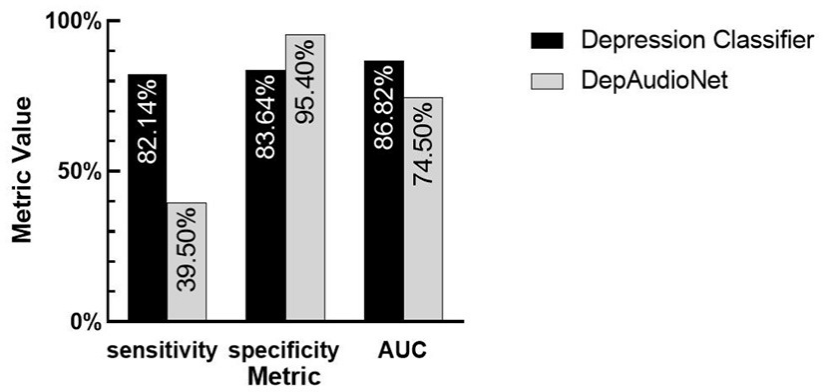
The comparison between two models. Depression classifier: traditional hand-engineered features model. DepAudioNet: deep learning-based Model. By observing the difference in sensitivity, specificity and AUC between the two models, the performance of pre-trained model is significantly improved can be seen.

In this section, we compare and discuss the classification performance between our baseline model built with traditional hand-engineered features (log-Mel spectrogram) against our depression classifier model. On the acoustic side, we observe a strong increase in all metrics compared to the baseline CNN-RNN model. The sensitivity especially benefits from the pre-training approach. These results not only highlight the increased robustness of our pre-training approach, but also the efficacy of the random sampling strategy that ensures a similar proportion of depressed and healthy examples during the training process. Indeed, the sensitivity and specificity are both at a comparable level shown in Figure 3, despite the highly unbalanced nature of the dataset.

### Relative analysis

**Figure 4:**
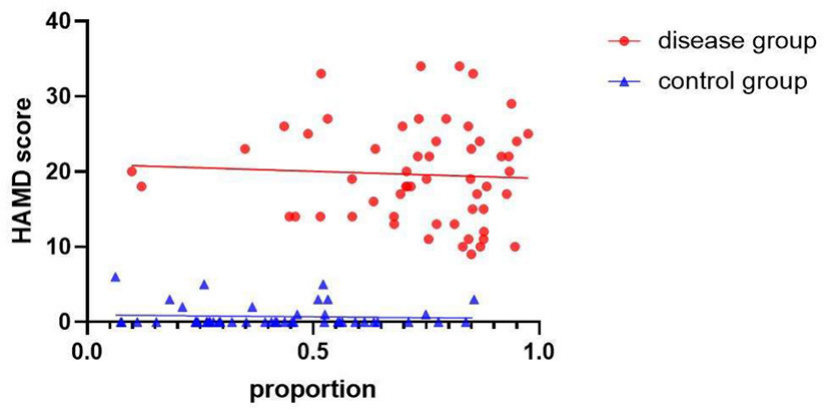
Correlation between proportion and HAMD score. Relationships between severity of MDD evaluated by HAMD score and proportion graded by AI models, for participants with MDD (*N* = 56, depression group) and healthy controls (*N* = 47, control group).

In order to screen the influencing factors related to the accuracy of the model, Spearman analysis was used to determine the correlation between the severity of the disease and the accuracy of the model, which is evaluated by proportion. The results showed in Figure 4 that there was no significant correlation between proportion and HAMD score (*r*_dep_ = −0.06, 95%CI: −0.31 to −0.21, *P*_dep_ > 0.05; *r*_con_ = −0.06, 95%CI: −0.34 to 0.23, *P*_con_ > 0.05, respectively).

### Depressive remission

**Figure 5:**
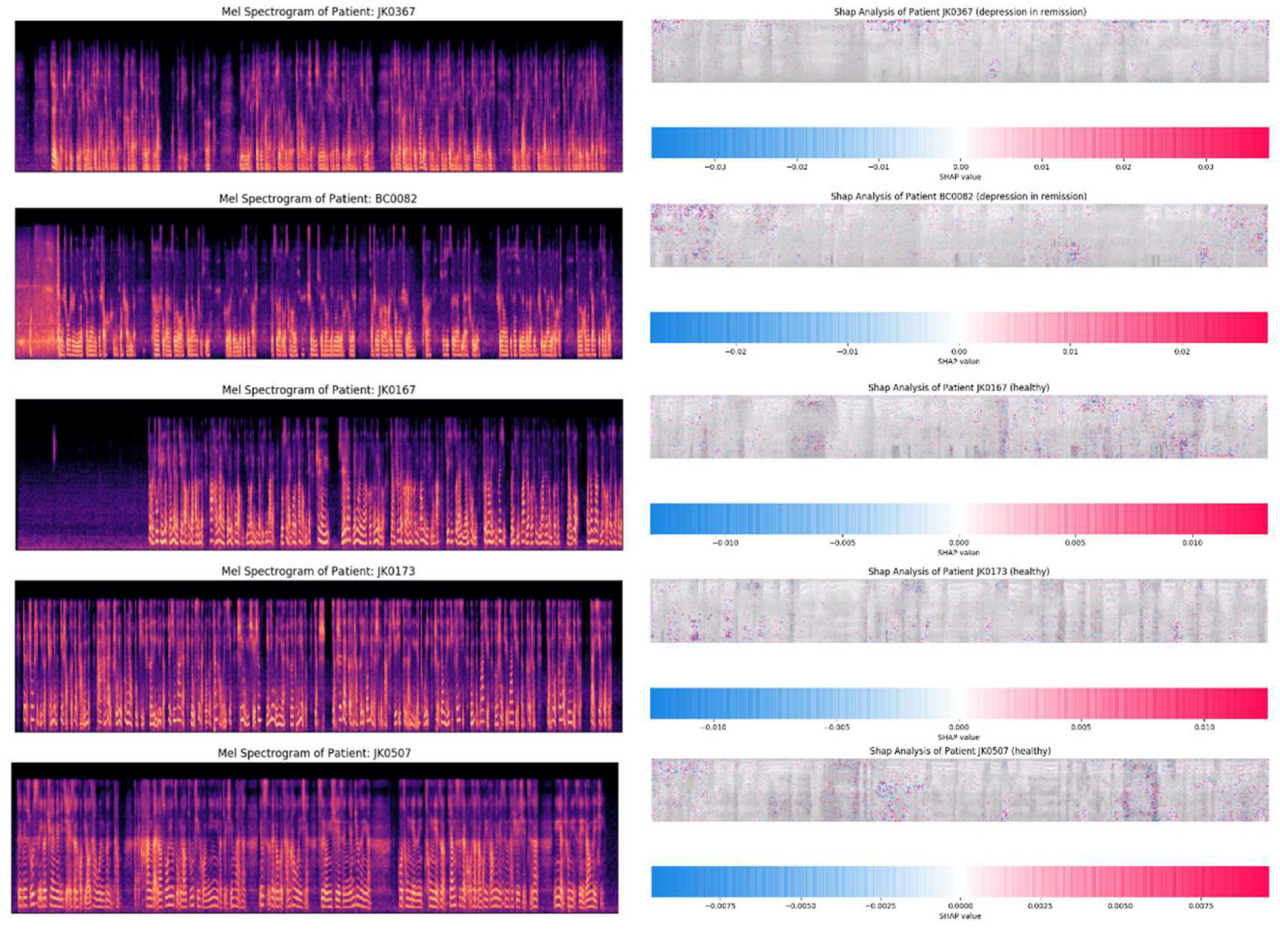
The mel spectrogram of participants in remission. Participants whose ID are JK0167, JK0173, JK0507, and JK0367 were diagnosed with major depressive disorder in remission by reference standard, and the first three were classified as healthy people by the depression classifier, and the latter as depression.

In the course of the experiment, we collected the speech information of four patients with depression in remission and predicted them using the model, of which three samples were identified as healthy control group, whose number is JK0167, JK0173, JK0507, respectively, and one as depression (JK0367). Their prediction scores are respectively 0.2455, 0.1865, 0.1836, and 0.8216. As Figure 5 shows, the Mel spectrograms of patients with major depressive disorder in remission look more similar to healthy control group, rather than that of depressive episodes.

Psychomotor retardation is a long-established component of depression. These psychomotor retardation manifestations include slowed speech, decreased movement, and impaired cognitive functions have different characteristics correlations with the level of severity of the patient, even though deep learning models can be used as automated classifiers for depression. It's important to build tools for explaining the predictions about what features the model learns during its training.

As an initial step for model interpretability in complex deep learning models we have used SHapley Additive exPlanations (SHAP) (35). SHAP values equals the difference between the expected model output (averaged over the background dataset) and the current model output. In a deep learning model for each feature SHAP assigns a feature importance value for a particular prediction at a pixel level where red pixels increase the model output while blue pixels decrease the output. This method gives us insight which areas of the spectrogram the model is learning most useful features with high predict power.

The SHAP analysis of the patients with depression in remission have similar spectral characteristics to healthy control group. Therefore, psychomotor retardation manifestations in these sub groups can be further explored with task specific deep learning models with improved machine learning techniques.

## Discussion

### Contributions and limitations

Screening and diagnosis is an important aspect of depressive disorders management cycle. If the relevant diagnosis can be made in a private and convenient environment, it will greatly promote patients to receive early treatment. The examination process of electroencephalogram and neuroimaging is relatively complex and requires advanced equipment, domain experts to analyze the results, which makes a lot of limitations in the practical application of the scene (36). The advancements in automated screening using such data still have limitations on practical applications as the cost per diagnosis is higher limiting the access for general public. In contrast, voice, as a common and objective measurement, can be collected in a relatively private environment efficiently. This enables researchers to conduct studies on longitudinal data on treatment effectiveness and better support the patients in the recovery journey. Vocal biomarker technology has high potential to become an important research vertical in the affective computing and depressive disorder recognition in recent years because of its simplicity and effectiveness.

Compared with previous studies, this study mainly has the following differences: (1) in the real environment, voice is collected through the subjects' smartphones, which means that there is more noise in the data samples; (2) the subjects are more targeted and focus on the effectiveness of the model in the middle-aged and elderly groups; (3) implementing robust machine learning method using self-supervised transfer learning approach; (4) exploring model interpretability in deep learning models.

When pre-processing the voice samples in the process of establishing model, we found that the depressive patients mainly came from outpatients, the voice collection places were mostly in the consultation room or ward, and the non-depressive patients mainly came from the network or non-medical scenes. Most of the places where sounds are collected are at home or in a quiet and empty room, which leads to a much higher proportion of voice sample noise and reverberation in depressed patients than in non-depressive patients. The model learns information other than biological speech information, and is more inclined to judge the samples with poor speech quality as depression samples. Therefore, in order to avoid voice quality affecting the performance of the model and deal with the problem of data imbalance, we tried to reduce noise, discard clips with poor sound quality (SNR ≤ 15), as well as other advanced audio augmentations including noise/reverb addition etc., and achieved the balance of signal-to-noise ratio between disease data and healthy data of training test in the training preprocessing stage. Implementing more complex model training architectures we have observed pretrained embeddings are much more robust than hand engineered features such as log Mel spectrograms therefore the model generalizes better for data in the real-world environment.

Meanwhile, some previous studies have shown that there is a certain correlation between pronunciation and emotion. Emotional changes may lead to acoustic changes, which means depression patients receiving different emotional stimuli may affect their performance in phonetic acoustic features. With emotional feature-related tasks in acoustic feature analysis, the accuracy of depression classification can be improved (37). On the other hand, different task paradigms will affect the feature extraction of speech, and non-fixed tasks are often more helpful to the two-classification of speech than fixed tasks. The speech data used in this study are all reading tasks, and the effects of different emotional titers on the accuracy of the model are not involved. The current sensitivity is 82.14% and specificity is 80.35%. In the further experiments, different task paradigms and emotional stimuli will be added to improve the performance of accuracy of the model, and explore the different effects of positive and negative emotions.

In addition to the accuracy of the model, we also pay attention to its stability. In order to test the stability of the model, we use different severity as covariables and the accuracy of the model as dependent variables. Correlation analysis was carried out. HAMD score was used as the criteria of evaluation of severity. The results suggest that there is no correlation between the accuracy of the model and the above covariables, and the performance is stable in different subgroups.

At the same time, in the course of the study, we observed that the model tends to classify the speech samples in the remission stage of depression into healthy control group, which is consistent with the results of previous studies: the speech feature distribution of patients in remission stage is more similar to that of healthy subjects, and there is a gap between the depression episode individual and the remission individual (32).

### Future works

Considering that it is difficult to verify the statistical differences in the current amount of remission data, to verify the above hypotheses, our future research process will consider collecting more speech data of participants in depressive remission and use the model to classification.

We also have developed model interpretability technique to broaden our understanding which areas the model is learning the most impactful features, Analyzing the time stamps for false positives and false negatives provides insights whether model has learned information other than biological speech characteristics and further the acoustic variations of tasks which have the highest prediction power differentiating healthy and depressed speech samples. In addition, the interpretability of the model features enables the linguistics experts and psychoacoustic experts to design better vocalization tasks to gather more powerful datasets.

We hope to address limitations of the work such as improving SNR estimation algorithms and employ more robust data augmentation techniques to reduce bias in datasets caused by acoustic differences in samples caused by environmental noise. Developing model interpretability techniques also enables us to have much deeper collaborations with psychoacoustic experts to further explore the effect of psychomotor retardation in respect to levels of depression.

In conclusion, the main work of this study is to combine artificial intelligence with speech, through deep learning, to extract the objective differences of acoustic signals between patients with depression and ordinary people who is Mandarin-speaking, and to test their effectiveness in the elderly groups, to improve the method of assistant diagnosis of depression in clinical scene.

## Data availability statement

The original contributions presented in the study are included in the article/Supplementary material, further inquiries can be directed to the corresponding author/s.

## Ethics statement

The studies involving human participants were reviewed and approved by the Ethics Committee of the Sixth Hospital of Peking University. The patients/participants provided their written informed consent to participate in this study. Written informed consent was obtained from the individuals for the publication of any potentially identifiable images or data included in this article.

## Author contributions

WY and YLin designed the study and submitted the study design for ethic commission of the Sixth Hospital of Peking University. BL, ZZ, and QW serving as the technical support coordinator for the study. YS, TL, YLin, CS, and YLia contributed to data collection. BL and YLin wrote the first draft of the manuscript. All authors contributed to and have approved the final manuscript.

## Funding

This study was supported by the National Key R&D Program of China (2021YFF1201103 and 2016YFC1307000), National Natural Science Foundation of China (81825009), Collaborative Research Fund of Chinese Institute for Brain Research Beijing (2020-NKX-XM-12), CAMS Innovation Fund for Medical Sciences (2021-I2M-C&T-B-099 and 2019-I2M-5-006), and PKUHSC-KCL Joint Medical Research (BMU2020KCL001).

## Conflict of interest

Authors BL, ZZ, and QW were employed by company Voice Health Tech. The remaining authors declare that the research was conducted in the absence of any commercial or financial relationships that could be construed as a potential conflict of interest.

## Publisher's note

All claims expressed in this article are solely those of the authors and do not necessarily represent those of their affiliated organizations, or those of the publisher, the editors and the reviewers. Any product that may be evaluated in this article, or claim that may be made by its manufacturer, is not guaranteed or endorsed by the publisher.
